# VAF–tumor content graph: a simple visual framework for interpreting hereditary cancer variants and supporting genetic counseling in tumor-only sequencing

**DOI:** 10.1038/s10038-026-01494-7

**Published:** 2026-07-29

**Authors:** Mina Kashima, Hiroshi Tsubamoto, Tomoko Ueda, Chinatsu Kinjo, Chiho Okada, Yoshiko Muroi, Mako Ueda, Taiichiro Otsuki, Kozo Kataoka, Masayuki Nagahashi, Ikuo Matsuda, Hideaki Sawai, Takashi Kijima, Ayako Miyazaki

**Affiliations:** 1 Department of Clinical Genetics, Hyogo Medical University Hospital, Nishinomiya, Japan; 2 Department of Obstetrics and Gynecology, Hyogo Medical University School of Medicine, Nishinomiya, Japan; 3 Department of Obstetrics and Gynecology, Takarazuka City Hospital, Takarazuka, Japan; 4 Oncology Center, Hyogo Medical University Hospital, Nishinomiya, Japan; 5 Department of Respiratory Medicine, Hyogo Medical University School of Medicine, Nishinomiya, Japan; 6 Department of Gastroenterological Surgery, Division of Lower Gastrointestinal Surgery, Hyogo Medical University School of Medicine, Nishinomiya, Japan; 7 Department of Breast and Endocrine Surgery, Hyogo Medical University School of Medicine, Nishinomiya, Japan; 8 Department of Breast and Endocrine Surgery, Nagoya University Graduate School of Medicine, Nagoya, Japan; 9 Department of Surgical Pathology, Hyogo Medical University School of Medicine, Nishinomiya, Japan

**Keywords:** Genetics research, Cancer genomics

## Abstract

Comprehensive genomic profiling (CGP) using tumor-only sequencing detects pathogenic or likely pathogenic (P/LP) variants in hereditary cancer susceptibility genes (HCSGs). However, interpreting the biological origin and clinical significance of detected variants is often challenging, complicating communication and decision-making during genetic counseling. We developed a variant allele frequency (VAF)–Tumor Content Graph as a simple visual framework that integrates VAF and tumor content with theoretical reference lines based on the Knudson two-hit hypothesis to support variant interpretation and clinical discussion. We retrospectively reviewed patients who underwent CGP using both tumor-only and tumor–normal paired panels between 2018 and 2025. P/LP variants in HCSGs recommended for disclosure by the institutional expert panel were plotted on the graph. Among 103 patients, 35 were confirmed to have germline P/LP variants. Among *BRCA1/2* variants, LOH was observed in 12 of 22 hereditary breast and ovarian cancer (HBOC)-associated tumors and in 2 of 5 non-HBOC tumors. Among other HCSGs, four of eight cases harbored two P/LP variants distributed along theoretical lines corresponding to germline and somatic alterations. Overall, 18 of 35 cases (51%) showed patterns consistent with the two-hit model. In tumors with mismatch-repair deficiency in one patient and *POLE* mutations in two patients, multiple variants clustered along the somatic line. The VAF–Tumor Content Graph provides a practical visual framework for interpreting HCSG variants by illustrating potential germline or somatic origin and underlying tumorigenic mechanisms, and may facilitate communication and shared decision-making during genetic counseling.

## Introduction

Comprehensive genomic profiling (CGP) is widely used in oncology, and tumor-only panels are more commonly performed than tumor–normal paired analyses. Although CGP is primarily designed to identify actionable mutations, it frequently detects variants in HCSGs. Patients referred for genetic counseling after presumed germline pathogenic or likely pathogenic variants (GPVs) are identified through tumor-only testing often have advanced or progressive disease. Meanwhile, insurance coverage for germline testing remains highly limited, making confirmatory testing both psychologically and financially burdensome. In this setting, helping clinicians and patients interpret and discuss the likely origin of detected variants is of great clinical importance for appropriate genetic counseling and for the efficient use of limited medical resources.

Interpretation varies internationally. The European Society for Medical Oncology (ESMO) and Japanese guidelines recommend the use of variant allele frequency (VAF) thresholds for confirmatory germline testing [1, 2], whereas the American Society of Clinical Oncology (ASCO) and the National Comprehensive Cancer Network (NCCN) do not adopt VAF-based thresholds [3]. Moreover, hypermutated tumors—such as those with mismatch repair (MMR) deficiency or *POLE* mutations—yield multiple variants, complicating the interpretation.

Therefore, we devised the VAF–Tumor Content Graph, a simple visual framework integrating VAF and tumor content to support interpretation of tumor-only sequencing results based on Knudson’s two-hit hypothesis. Rather than serving as a standalone classifier, the graph is intended to facilitate clinical reasoning and communication during genetic counseling by visually illustrating possible biological mechanisms underlying detected variants. As a complementary tool to existing guideline-based assessment, the utility of this approach in gynecologic cancers has previously been demonstrated [4]. In that study, visualization of patterns consistent with biallelic inactivation also provided supportive information for therapeutic decision-making for treatments targeting such alterations, including PARP inhibitors or immune checkpoint inhibitors. The present study extends this concept to a broader pan-tumor setting, illustrating how the graph can visualize the potential germline or somatic origin of detected variants and the underlying two-hit mechanisms across diverse tumor types to support interpretation and communication during genetic counseling.

## Materials and methods

### Study design and ethics

This retrospective study was approved by the Institutional Review Board of Hyogo Medical University (no. 5152), and was conducted at Hyogo Medical University. All the participants provided written informed consent in accordance with the Declaration of Helsinki.

### Patients

Patients who underwent CGP between August 2018 and September 2025 and were referred to the institutional Molecular Tumor Board (MTB) were included. Clinical and genomic data, including age, cancer type, tumor content, and variant details, were extracted from electronic medical records.

## Disclosure and counseling

The Japanese disclosure criteria were changed between the initial and revised versions of the national guideline. Briefly, the 2019 guidelines recommended confirmatory germline testing for PGPVs in *BRCA1/2* regardless of VAF, whereas the 2021 revision required a VAF ≥ 10% for confirmatory testing. Variants in *MLH1*, *MSH2*, *MSH6*, and *PMS2* with a VAF ≥ 10%, as well as other genes meeting the VAF thresholds (≥30% for single-nucleotide variants and ≥20% for insertions/deletions), were also recommended for further evaluation[2].

Based on these guidelines, the molecular tumor board—with the attending physician present—identified hereditary cancer susceptibility genes requiring disclosure. The attending physician communicated the result to the patient. At our institution, patients were provided with easy access to genetic counseling: they could speak directly with a genetic counselor free of charge on the same day, or, if preferred, schedule a counseling appointment later by telephone.

### Analysis

**Fig. 1:**
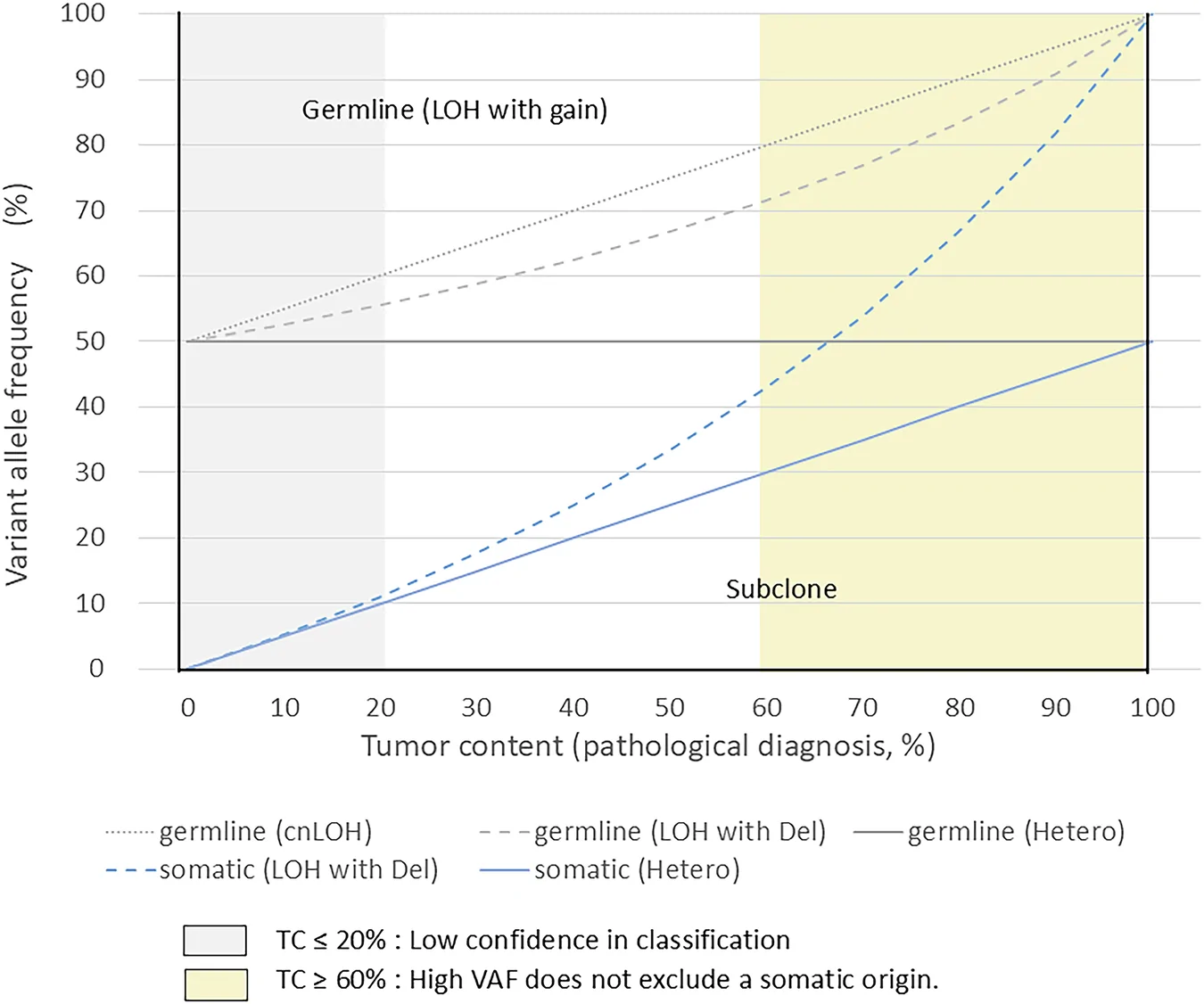
VAF–tumor content graph of variants detected by NGS-based tumor genomic profiling. The lines theoretically represent germline and somatic variants according to the two-hit mechanism described in Knudson’s hypothesis [13]. In most cases of HBOC, loss of the normal allele causes germline variant genes to be theoretically plotted along the dotted line representing “germline (LOH with Del)” [14, 15]. When the affected variant allele was duplicated, germline variant genes were theoretically plotted along the dotted line representing “germline (cnLOH).” When the affected allele was amplified (≥2 copies), the VAF exceeded the dotted line representing “germline (cnLOH)” and plotted within the area of “germline (LOH with gain)“. Theoretical lines representing somatic variants with LOH due to deletion of the wild-type allele is also shown. germline (LOH with gain), germline variants with loss of heterozygosity (LOH) and copy gain of the variant allele; germline (cnLOH), germline variants with copy-neutral LOH (uniparental disomy); germline (LOH with Del), germline variants with LOH due to deletion of the wild-type allele; germline (Hetero), heterozygous germline variants; somatic (LOH with Del), somatic variants with LOH due to deletion of the wild-type allele; somatic (Hetero), heterozygous somatic variants; Subclone: subclonal variants in the tumor

Both presumed and confirmed GPVs were plotted on the VAF–Tumor Content Graph, which was modified from our previous report (Fig. 1) [4]. In this graph, tumor content is plotted on the x-axis and VAF on the y-axis. Tumor content was estimated by pathological assessment of tumor cellularity, which was adopted because the tumor content determined by pathological evaluation at our institution was consistent with that reported by FoundationOne CDx and showed better agreement with the theoretically estimated lines than tumor purity calculated from NGS data.

The graph incorporates theoretical reference lines derived from the Knudson two-hit hypothesis, including a line representing loss of heterozygosity (LOH) and lines corresponding to expected patterns for germline and somatic variants. For each case, detected PGPVs or GPVs recommended for disclosure by the MTB were plotted according to their observed VAF and tumor content.

The plotted positions of variants were visually compared with theoretical lines and areas derived from the two-hit hypothesis to determine whether they showed patterns consistent with the two-hit model. We further evaluated the proportion of patients whose variants aligned with these theoretical patterns. In addition, in non-hereditary tumors with mismatch-repair deficiency or *POLE* mutations, which typically harbor numerous somatic variants, including variants annotated as pathogenic or likely pathogenic (P/LP) in HCSGs, we examined whether multiple detected variants clustered along the somatic reference line. This analysis allowed assessment of whether such tumors could be visually distinguished from cases with potential germline variants. Overall, this evaluation was performed to explore the utility of the graph as a supportive visual tool for interpreting variants and facilitating genetic counseling.

Descriptive statistics were performed using Prism 9 software (GraphPad Software, La Jolla, CA, USA).

## Results

**Fig. 2:**
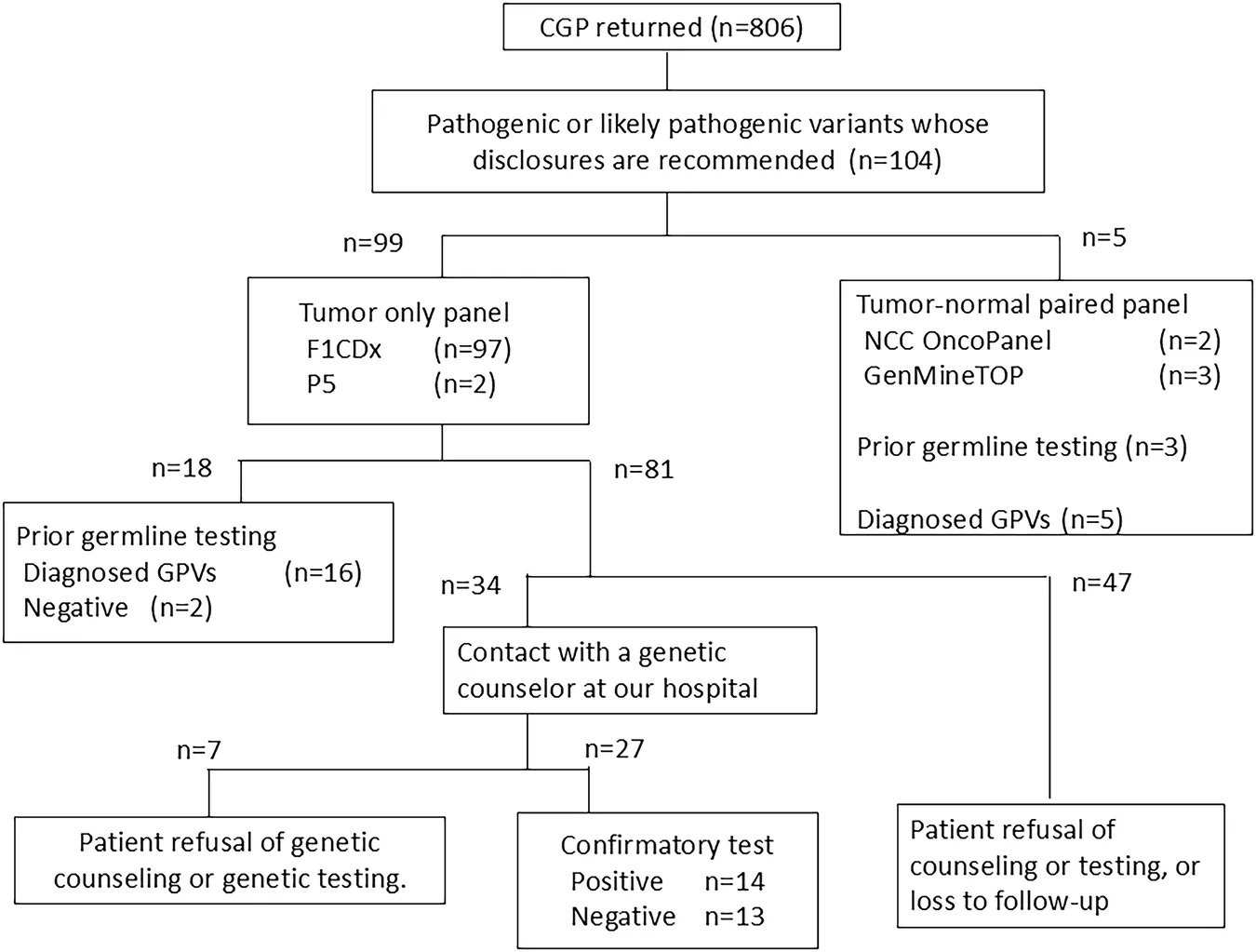
Patient flow diagram. Between Nov. 2018 and Sep. 2025, genomic sequencing of cancer tissues with or without blood testing was reported in among 806 patients. Twenty-seven patients (33%) underwent confirmatory tests among the 81 patients who received tumor only panels and whose prior germline testing was not conducted. After CGP testing, germline pathogenic or likely pathogenic variants were newly diagnosed in 16 patients (13%) among those had GPV. CGP comprehensive genomic profiling, F1 CDx FoundationOne CDx, GPVs germline pathogenic and likely pathogenic variants

Among the 806 patients who underwent CGP, 103 (12.8%) carried P/LP variants in the HCSGs (Table 1). Ninety-nine patients had tumor-only panels, and five underwent tumor–normal paired testing. Thirty-five patients were confirmed to have GPVs—19 identified by prior germline testing before CGP and 16 were newly diagnosed after CGP—whereas 13 were confirmed to be negative and 47 were untested or lost to follow-up (Fig. 2). Among the 35 patients with confirmed GPVs, one of three patients with familial adenomatous polyposis was clinically diagnosed, and one patient with fumarate hydratase–deficient renal cell carcinoma was pathologically confirmed by immunohistochemistry.

**Table 1:** Patient characteristics

		Germline findings of CGP for disclosure	GPV confirmed
Number of patients		103	35
Gender			
	Male	34	4
	Female	69	31
Age (year old)			
	Median	60	58
	Oldest	84	79
	Youngest	22	22
Tumor types			
	Breast cancer	20	6
	Ov	14	11
	Pancreatic	9	4
	BTC	9	0
	EC	8	4
	Mesothelioma	8	1
	Prostate	7	2
	CRC	7	4
	STS	5	1
	Cervical cancer	3	1
	RCC	1	1
	Others	12	
P/LP variants (max VAF)			
	*BRCA2*	27	17
	*BRCA1*	13	10
	*ATM*	9	0
	*BAP1*	8	0
	*CDH1*	5	0
	*APC*	3	3
	*MLH1*	3	0
	*MSH2*	3	2
	*MSH6*	3	1
	*NF1*	3	1
	*SDHA*	3	0
	*TP53*	3	0
	*BRIP1*	2	0
	*NF2*	2	0
	*SMAD4*	2	0
	*FH*	1	1
	others	13	
TMB (Muts/Mb)			
	<10	87	31
	10–100	14	4
	>100	2	0

*CGP* comprehensive genomic profiling, *GPV* germline pathogenic or likely pathogenic variants, *VAF* variant allele frequency, *P/LP* pathogenic or likely pathogenic, *TMB* tumor mutation burden

**Fig. 3:**
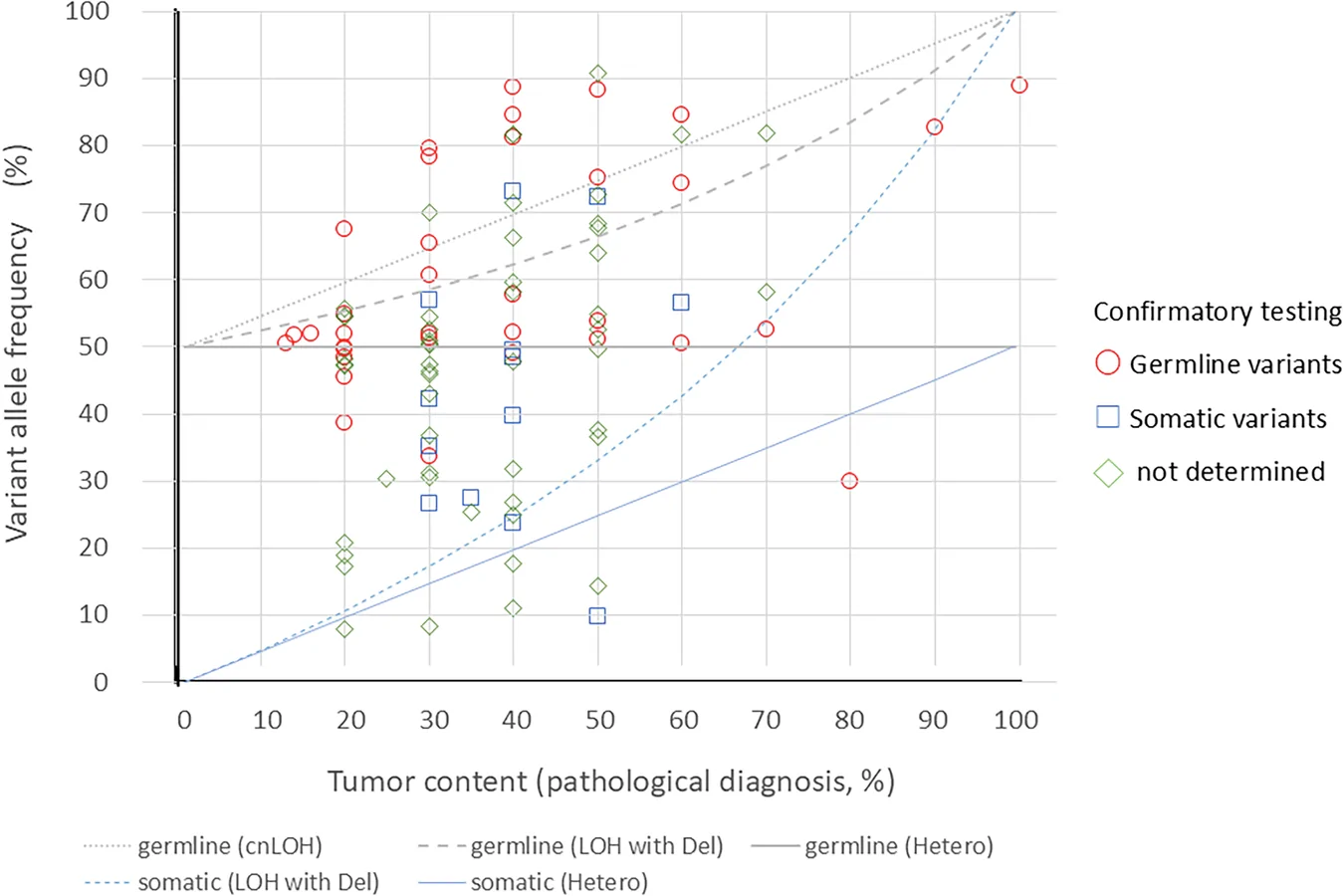
Susceptible hereditary cancer genes recommended for disclosure plotted on the VAF–Tumor Content Graph. Variants in genes recommended for disclosure, identified using both tumor-only and tumor–normal paired panel analyses, were plotted (*n* = 100). Only single-nucleotide variants and insertion–deletions (indels) were included. Variants without calculable variant allele frequency (VAF), including exon deletions (loss of exon 3, exons 3–31, and exons 11–27 in three patients, respectively), were excluded. For each patient, the gene with the highest VAF among those recommended for disclosure was selected for plotting. Germline and somatic variants were confirmed in 35 and 13 patients, respectively, while 52 patients were classified as undetermined due to refusal of genetic counseling or testing, or loss to follow-up

**Fig. 4:**
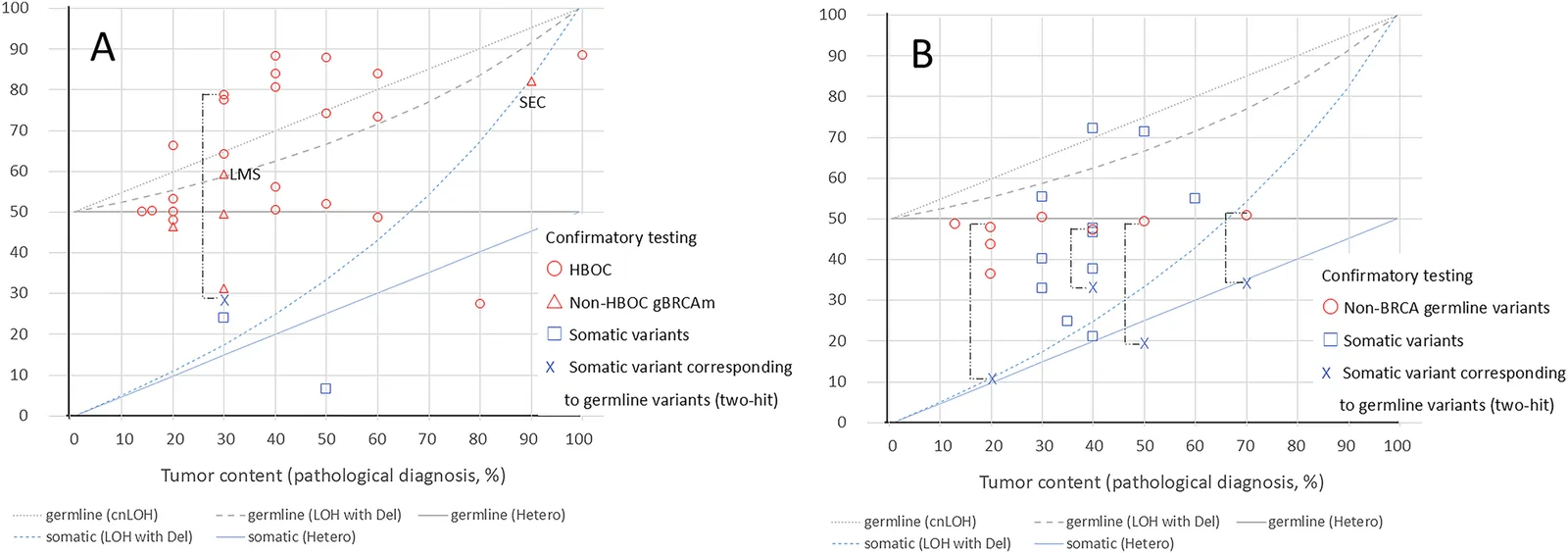
Germline-derived pathogenic or likely pathogenic variants identified by genomic sequencing of cancer tissues. **A** *BRCA1/BRCA2* pathogenic or likely pathogenic variants (*n* = 27). **B** Pathogenic variants in genes other than *BRCA1/BRCA2* (*n* = 8). **A** Germline-derived pathogenic or likely pathogenic variants in *BRCA1* and *BRCA2* were identified in 9 and 18 patients, respectively. Hereditary breast and ovarian cancer (HBOC) cases included ovarian cancer (*n* = 10), breast cancer (*n* = 6), pancreatic cancer (*n* = 4), and prostate cancer (*n* = 2). A somatic pathogenic variant corresponding to the germline *BRCA2* variant was also identified in one patient. Five patients had non-HBOC cancer. Serous endometrial carcinoma (SEC) and uterine leiomyosarcoma (LMS) located along the line representing loss of heterozygosity with deletion showed long-term clinical responses, responding to olaparib and trabectedin, respectively. The SEC case has been previously reported [8], and the LMS case has maintained a durable response for three years. Both drugs are indicated for tumors with biallelic alterations in *BRCA2*. **B** Genomic sequencing of the cancer tissues revealed both germline and somatic variants consistent with Knudson’s two-hit hypothesis (*n* = 4). One of three patients with familial adenomatous polyposis was clinically diagnosed. HBOC hereditary breast and ovarian cancer, LMS uterine leiomyosarcoma, SEC serous endometrial carcinoma, gBRCAm germline *BRCA* mutations/variants

Most confirmed GPVs (34/35; 97%) exhibited a VAF ≥ 30%, and only one case had a VAF of 29% (Fig. 3). Among the 27 patients with *BRCA1/2* variants, 22 had hereditary breast and ovarian cancer (HBOC)–related malignancies and five had non-HBOC cancers (Fig. 4A). LOH was inferred in 12 of 22 (55%) HBOC cases and in two of five non-HBOC cancers, both of which responded to olaparib and trabectedin, drugs indicated for tumors with biallelic alterations in *BRCA2*. Among the eight patients with GPVs in other genes (e.g., *MSH2*, *MSH6*, *FH*, *NF1*, *APC*), biallelic inactivation involving both germline and somatic variants was inferred in four cases (Fig. 4B). Overall, 18 of 35 cases (51%) showed patterns consistent with the two-hit model.

**Fig. 5:**
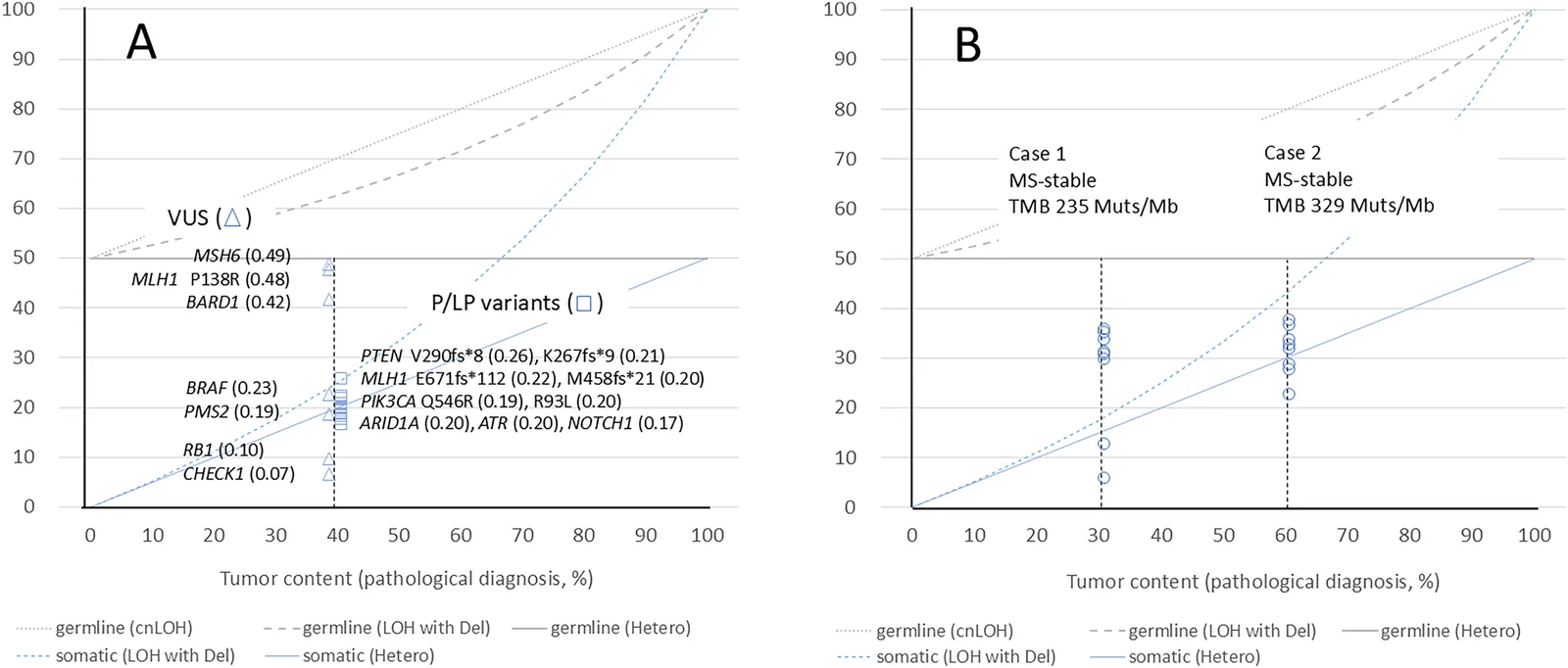
Representative cases of hypermutated tumors identified through tumor sequencing. **A** dMMR, non-Lynch tumor, **B** *POLE*-mutated tumor. **A** Immunohistochemistry demonstrated loss of *MLH1* and *PMS2* protein expression, consistent with mismatch repair deficiency (dMMR). Comprehensive genomic profiling using FoundationOne CDx™ revealed microsatellite instability–high (MSI-H) status and a tumor mutation burden (TMB) of 33 muts/Mb. Germline multigene panel testing identified variants of uncertain significance (VUS) in *MSH6*, *MLH1*, and *BARD1*, derived from the germline with variant allele frequencies (VAFs) of 0.49, 0.48, and 0.42, respectively; no germline pathogenic or likely pathogenic variants were detected. When multiple mutations were identified within the same gene, the predicted protein effect of each variant is shown, and VAFs are indicated in parentheses following each gene symbol. **B** Ultra-mutated tumors exhibited multiple pathogenic or likely pathogenic (P/LP) variants in hereditary cancer-susceptibility genes, occasionally including more than one variant within the same gene. Each P/LP variant is indicated by an open circle. Germline multigene panel testing in Case 1 revealed no pathogenic variants. *POLE* DNA polymerase epsilon, TMB tumor mutational burden, muts/Mb mutations per megabase

Sixteen patients exhibited a high tumor mutation burden (≥10 mutations/Mb), and seven underwent germline testing, and four were confirmed to have hereditary cancers. In the case of mismatch-repair–deficient endometrial carcinoma with a tumor mutation burden of 33 mutations/Mb, numerous variants were identified; however, except for three variants of uncertain significance in *MSH6*, *MLH1*, and *BARD1*, all were determined to be of somatic origin (Fig. 5A). Two *POLE*-mutated endometrial carcinomas (≥200 mutations/Mb) displayed variant clustering along the somatic line, which was consistent with somatic hypermutation (Fig. 5B).

## Discussion

This study is the first to apply the VAF–Tumor Content Graph in a pan-tumor context. The VAF–Tumor Content Graph visualizes the relationship between VAF and tumor content, serving as a practical visual framework for interpreting the potential germline or somatic origin of detected variants. Importantly, its primary value lies in providing an intuitive conceptual framework that supports clinical interpretation and facilitates communication between healthcare professionals and patients during genetic counseling, rather than replacing existing diagnostic algorithms. In our cohort, germline confirmation was exceedingly rare when the VAF was <30%, which is consistent with previous reports [5]. In the present study, plotting VAF against tumor content on a single graph enabled visualization of theoretical patterns consistent with loss of heterozygosity (LOH) and other biallelic (“two-hit”) events involving germline and somatic variants. We observed patterns compatible with copy-neutral LOH and LOH with copy gain.

In Japanese clinical guidelines, when patients present with phenotypic features—such as current illness, past medical history, or family history—closely associated with detected HCSGs, disclosure and referral for genetic counseling are recommended regardless of VAF. In contrast, for PGPVs without characteristic phenotypic features, VAF serves as an important criterion for determining whether disclosure and genetic counseling should be recommended [1]. Theoretically, germline variants are expected to show a VAF of approximately ≥50%, and in clinical practice, variants with VAF values ≥ 20–30% are generally considered for disclosure.

However, VAF can also increase in somatic variants accompanied by LOH, particularly with increasing tumor content and copy number gain of the variant allele. During the initial development of this graph, we omitted theoretical lines representing somatic LOH, as variants with high VAF are more frequently recommended for germline testing in clinical practice (with some exceptions, such as TP53), and inclusion of these lines made the graph visually complex, as described in our previous report [4]. Nevertheless, from a clinical perspective, the possibility that high-VAF variants may still be of somatic origin is an important consideration during genetic counseling. Therefore, in the present study, we incorporated theoretical lines representing somatic LOH into the revised VAF–Tumor Content Graph. In addition, we developed and publicly released software, the VAF–TC Visualizer, based on the VAF–Tumor Content Graph. The software generates a warning when TC is ≥60% and VAF is at or above the theoretical line representing somatic LOH with deletion, indicating a region where germline LOH and somatic LOH may overlap and are difficult to distinguish based on VAF alone.

*TP53* is a representative tumor suppressor gene that frequently undergoes biallelic inactivation through somatic LOH during tumorigenesis across various cancer types, and *TP53* mutations often show high VAF values in tumor sequencing data [6]. Consequently, VAF values exceeding 50% do not necessarily indicate germline origin in tumor-only sequencing. In tumor-only panel testing, rare de novo cases of Li–Fraumeni syndrome caused by germline *TP53* mutations must also be considered. In current Japanese clinical practice, recommendations for confirmatory germline testing for *TP53* variants are made based not only on VAF but also on clinical features including age at onset, personal history, and family history.

More than 40% of the study cohort consisted of HBOC-related tumors, which are known to have relatively high frequencies of LOH. Among non-HBOC tumors, *BRCA1/2* germline variants have occasionally been reported as passenger variants. However, although less frequent than in HBOC tumors, LOH-associated second hits have also been observed in non-HBOC tumors [7]. The number of non-HBOC tumors in the present study was limited; however, two cases suggestive of LOH-associated second hits showed marked responses to drugs targeting biallelic inactivation of *BRCA1/2* [8]. This finding supports the potential utility of the VAF–Tumor Content Graph for visualizing clinically relevant two-hit events across diverse tumor types.

Although hypermutated MMR-deficient and *POLE*-mutant tumors frequently display multiple variants in hereditary cancer susceptibility genes, only ~10–15% of dMMR tumors are attributable to Lynch syndrome [9, 10]. In such contexts, numerous somatic variants can obscure germline findings, and the VAF–Tumor Content Graph may assist clinicians in interpreting complex variant profiles.

Several computational models have been proposed to estimate the likelihood of PGPVs from tumor-only sequencing data. The Loss Of Heterozygosity Germline Inference Calculator (LOHGIC) algorithm has been reported to predict germline status using tumor sequencing results [11]. In an evaluation of 28 cases with available germline *BRCA1/2* testing, LOHGIC demonstrated 93% accuracy, 100% precision, and 96% recall for distinguishing germline from somatic variants. However, the clinical context of the analyzed cases, including whether the tumors were classical HBOC-associated cancers or non-HBOC/off-tumor types, was not clearly described. More recently, a computational prediction model was reported from Japan [12]. The model achieved a c-index of 0.96–0.97, suggesting high performance for predicting hereditary cancer susceptibility. However, these models do not directly determine whether the variant represents a biallelic alteration, which is one of the key predictive markers for therapeutic response to PARP inhibitors. In this context, visual approaches such as the VAF–Tumor Content Graph may provide additional insight into the likelihood of biallelic inactivation.

Most patients who undergo CGP have advanced-stage cancer. For these patients, confirmatory germline testing often provides limited direct benefit to themselves, whereas the main benefit lies in risk assessment and preventive care for family members. Consequently, some patients may hesitate to undergo germline testing because of emotional or financial concerns, particularly when testing is only partially covered by medical insurance. In this context, the VAF–Tumor Content Graph developed in this study, which is applicable across multiple tumor types, may help patients and clinicians better understand the biological context of detected variants, supporting shared decision-making regarding confirmatory germline testing and family risk assessment. This visualization may facilitate more informative discussions during genetic counseling and support patients in making better-informed decisions.

### Limitations

This single-institution retrospective study may limit the generalizability of the findings and introduce potential selection bias when applied in routine clinical practice. Tumor content assessment was semi-quantitative, and interpretation may be less reliable, particularly when tumor content is low. However, in such cases, the quality of tumor-derived DNA is often insufficient for CGP, and sequencing is frequently discontinued before analysis. Consistent with this limitation, cases with VAF < 20% were rare in our cohort. The software, “VAF–TC Visualizer,” incorporates a shaded gray area for tumor content ≤20% to indicate regions where discrimination between somatic and germline variants is less reliable and displays a “Low Confidence” warning when tumor content is ≤20%. In addition, the validation cohort was enriched for classical HBOC-associated cancers such as breast, ovarian, and pancreatic cancers. Therefore, the observed discrimination performance may partly reflect tumor types with well-characterized LOH patterns, and further evaluation across a broader spectrum of solid tumors is warranted.

Although rare, reverse LOH, in which the mutant allele is lost within tumor cells, may cause germline variants to appear at low VAF values and mimic somatic variants or sequencing noise. Furthermore, in tumors with complex karyotypes such as aneuploidy or whole-genome doubling, observed VAF values may deviate from theoretical expectations derived from diploid assumptions, potentially reducing interpretive accuracy. Confirmatory germline testing was incomplete in some cases.

To facilitate broader clinical adoption of this approach, prospective validation studies with standardized pathology assessment and uniform germline testing in multi-institutional settings will be necessary to establish its scientific validity and clinical safety.

## Conclusions

The VAF–Tumor Content Graph is a practical visual framework that helps patients and clinicians better understand the biological context of detected variants, supporting shared decision-making regarding confirmatory germline testing and family risk assessment. Visualization of the relationship between VAF and tumor content may improve variant interpretation and facilitate communication during genetic counseling, while complementing existing guideline-based approaches. Future studies integrating visual tools such as the VAF–Tumor Content Graph with computational prediction models may further improve interpretation of tumor-only sequencing and support more precise clinical decision-making in genetic counseling and targeted therapy.

## Data Availability

To facilitate practical use in paper-based settings and allow easy customization, we provided the Excel file used to generate Fig. 1 and made it publicly available on GitHub (https://github.com/Clinical-Genetics-Suite/vaf-tc-app). The Excel file includes additional theoretical lines, including those assuming somatic variants with LOH and total copy numbers of 2, 3, and 4. We also developed a novel software tool, the “VAF–TC Visualizer,” an interactive web-based application designed to visually support interpretation of variant patterns and facilitate discussions during genetic counseling. In this software, automated alerts are generated such that when TC ≤ 20%, a warning is issued indicating reduced reliability of the theoretical lines, whereas when TC ≥ 60%, a message is displayed stating that “high VAF does not exclude a somatic origin,” reflecting the possibility that variants may arise from somatic LOH in addition to germline alterations. The application is available at https://vaf-tc-app.streamlit.app/.
